# Brain and spinal cord paired stimulation coupled with locomotor training facilitates motor output in human spinal cord injury

**DOI:** 10.3389/fneur.2022.1000940

**Published:** 2022-10-13

**Authors:** Timothy S. Pulverenti, Morad Zaaya, Ewelina Grabowski, Monika Grabowski, Maria Knikou

**Affiliations:** ^1^Klab4Recovery Research Program, The City University of New York, New York, NY, United States; ^2^PhD Program in Biology and Collaborative Neuroscience Program, Graduate Center of the City University of New York and College of Staten Island, New York, NY, United States; ^3^Department of Physical Therapy, College of Staten Island, The City University of New York, New York, NY, United States

**Keywords:** locomotor training, paired associative stimulation (PAS), rehabilitation, spinal cord injury (SCI), transspinal stimulation, transspinal evoked potentials (TEPs), input-output, recruitment order

## Abstract

Combined interventions for neuromodulation leading to neurorecovery have gained great attention by researchers to resemble clinical rehabilitation approaches. In this randomized clinical trial, we established changes in the net output of motoneurons innervating multiple leg muscles during stepping when transcranial magnetic stimulation (TMS) of the primary motor cortex was paired with transcutaneous spinal (transspinal) stimulation over the thoracolumbar region during locomotor training. TMS was delivered before (TMS-transspinal) or after (transspinal-TMS) transspinal stimulation during the stance phase of the less impaired leg. Ten individuals with chronic incomplete or complete SCI received at least 20 sessions of training. Each session consisted of 240 paired stimuli delivered over 10-min blocks for 1 h during robotic assisted step training on a motorized treadmill. Body weight support, leg guidance force and treadmill speed were adjusted based on each subject's ability to step without knee buckling or toe dragging. Most transspinal evoked potentials (TEPs) recorded before and after each intervention from ankle and knee muscles during assisted stepping were modulated in a phase-dependent pattern. Transspinal-TMS and locomotor training affected motor neuron output of knee and ankle muscles with ankle TEPs to be modulated in a phase-dependent manner. TMS-transspinal and locomotor training increased motor neuron output for knee but not for ankle muscles. Our results support that targeted brain and spinal cord stimulation alters responsiveness of neurons over multiple spinal segments in people with chronic SCI. Noninvasive stimulation of the brain and spinal cord along with locomotor training is a novel neuromodulation method that can become a promising modality for rehabilitation in humans after SCI.

## Introduction

Spinal cord injury (SCI) is characterized by hyperreflexia, a pathological epiphenomenon that can produce significant muscle force output. However, hyperreflexia coincides with unstable depolarization of spinal motoneurons and thus firing at a frequency and amplitude that cannot generate functional relevant muscle contractions (1). Hence, rehabilitation interventions that strengthen motor neuron output after SCI are in great need.

A traditional rehabilitation intervention is that of locomotor training with body weight support (BWS) and manual or robotic assistance of leg movements on a motorized treadmill (2, 3). Locomotor training improves muscle activation patterns and lower extremities motor scores, increases amplitude of muscle activity, and improves walking speed and distance (2, 4–7). These improvements likely are due to axonal re-growth, sprouting proximal to the lesion site, outgrowth of the corticospinal tract, decrease in muscle atrophy, and an increase in electromyographic (EMG) activity (8–10).

One potential supplementary intervention to locomotor training is stimulation of the central nervous system. Transcranial magnetic stimulation (TMS) over the somatosensory cortex paired with peripheral nerve stimulation (11–16) produces neurophysiological changes based on paired associative stimulation (PAS) induced plasticity mechanisms including long-term potentiation and long-term depression (LTP; LTD) (17, 18). LTP usually occurs when presynaptic inputs arrive before the postsynaptic inputs and LTD when these inputs arrive at a reverse order. LTD is also induced by low-frequency presynaptic stimulation, while LTP is induced by low frequency postsynaptic stimulation or high frequency presynaptic stimulation (19, 20). We have recently shown that transspinal stimulation over the thoracolumbar region uses similar neuronal pathways to those that convey descending motor drive onto spinal motor neurons and ascending sensory inputs to the sensorimotor cortex. This thesis is supported by the summation or depression of motor evoked potentials (MEPs), transspinal evoked potentials (TEPs), and soleus H-reflexes and M-waves on the surface EMG based on the relative timing of the stimuli targeting the brain and spinal cord (21, 22). Based on these findings we have theorized that transspinal stimulation induces neuromodulation across broad neural networks. When transspinal stimulation is delivered repeatedly as a rehabilitation intervention spasticity, hyperreflexia, and ankle clonus decreases, leg muscle activity increases during stepping, and motor neuron output increases when assessed by the recruitment input-output curves of TEPs recorded simultaneously from several muscles bilateral (23–26).

In this study we established changes in the net motor neuron output when TMS (presynaptic) was delivered before or after transspinal (postsynaptic) stimulation during locomotor training. We hypothesized that when transspinal stimulation is delivered before TMS motor neuron output increases in a phase-dependent manner leading to a more physiological motor activity during stepping. We further hypothesized that when transspinal stimulation is delivered after TMS motor output would minimally change. Motor neuron output was assessed *via* the phase-dependent modulation pattern of TEPs recorded bilaterally from knee and ankle muscles during assisted stepping in people with chronic SCI. The results reported here are for the same patients that we recently published on the reorganization of soleus H-reflex and flexion reflex excitability after the same intervention (27, 28).

## Materials and methods

### Subjects

Ten individuals with SCI with a mean age of 43.2 ± 15.2 years, height 175.0 ± 8.6 cm, and weight 78.0 ± 16.9 kg (mean ± SD) participated in the study. Clinical trial inclusion criteria were: age 18–75 years, chronic (>12 months) C2-T11 SCI, and presence of Achilles tendon reflexes. Exclusion criteria were the following: presence of supraspinal lesions, neuropathies of the peripheral nervous system, presence of pressure sores, presence of medical implants (e.g., cochlear implants, baclofen pumps etc.), presence of implanted metals that were not MRI-safe, degenerative neurological disorders, history of seizures, and leg bone mineral density T score < 1.5. To examine spinal circuit reorganization with minimal descending input, people with AIS A were included. Participants were asked to refrain from caffeine, alcohol, strenuous exercise for 12 h, and recreational drugs for 2 weeks before the initial testing session. Verbal and written informed consents were collected from all participants before study enrollment and participation. All experimental protocols were performed in compliance with the Declaration of Helsinki. The experimental protocols were approved by the Institutional Review Board (IRB) of the City University of New York (IRB No: 2017-0261) and registered on ClinicalTrials.gov (NCT04624607).

### Design

The clinical trial was a single-blind randomized design. On average participants completed 26.1 (±4.7) 1-h locomotor training sessions with TMS paired with thoracolumbar transspinal stimulation both delivered during assisted stepping in a robotic gait orthosis system (Lokomat 6 Pro^®^, Hocoma, Switzerland). Participants were randomized to receive either transspinal-TMS (*N* = 7) or TMS-transspinal (*N* = 6) PAS (Table 1). A computer generated simple-randomization sequence was used to create a random list of transspinal-TMS and TMS-transspinal interventions. As participants were recruited, they were assigned the next available intervention. Three participants completed both interventions with a 6-month washout period (identified in Table 1), in whom the second intervention was not randomized. The other participants were unable to participate in both interventions. Participants were blind to the PAS protocol and could not distinguish the order of stimuli because of the small interstimulus interval (ISI) between TMS and transspinal stimulation. Each participant completed experimental testing sessions at least 1 day before starting the intervention and then again, the day after the last training session. For the same subjects, we have recently reported on the soleus H-reflex and flexion reflex neurophysiological changes (27, 28) as primary outcomes of the clinical trial.

**Table 1:** Participant characteristics.

**Subject ID**	**Gender**	**Age (yrs)**	**Height (cm)**	**Weight (kg)**	**Injury level**	**AIS scale**	**# of training sessions**	**Cause of injury**	**Medication**
**Transspinal-transcranial**									
LR01^**a**^	M	31	185	59	C4-5	D	30	MVA	None
LR04^b^	M	60	170	91	C5-6	C	20	Fall	ASA 81 mgxD; Oxybotin 15 mg 2xD; Pravachol 40 mg xD; Pericolace 2xD
LR05	F	33	167	82	T12	A	19	MVA	Gabapentin 800 mg 3xD; Tramadol 2 50 mg 2xD; Amitriptyline 25 mg xD
LR06^**c**^	M	38	176	87	T11	D	30	GSW	Gabapentin; Percocet
LR07	M	57	181	115	C4	C	30	Fall	Gabapentin 300 mg 2xD; Oxybutynin 10 mg 3xD, Baclofen 10 mg 4xD; Oxycontin 10 mg; Ducolax 3xD; Senecot 3xD
LR09	M	37	181	84	C5	B	20	Diving	None
Mean	6M, 2F	43.3	175.3	80.0			24.8		
SD		15.8	7.3	18.5			5.1		
**Transcranial-transspinal**									
LR11^**a**^	M	31	185	59	C4-5	D	30	MVA	None
LR12^**c**^	M	38	176	87	T11	D	30	GSW	Gabapentin; Percocet
LR14	M	27	189	79	T8	A	20	MVA	Oxybutynin
LR15^**b**^	M	61	170	91	C5-6	C	23	Fall	ASA 81mgxD; Oxybotin 15mg 2xD; Pravachol 40 mg xD; Pericolace 2xD
LR20	F	57	160	64	C4-5	C	26	Surfing accident	None
LR21	M	71	172	64	C7	C	31	Fall	Gabapentin; Oxybutynin
Mean	5M, 1F	47.5	175.3	74.0			26.6		
SD		16.4	9.6	12.3			4.1		

Injury level corresponds to the neurological level of injury. The American Spinal Cord Injury Impairment Scale (AIS) is indicated for each subject based on sensory and motor evaluation per AIS guidelines. The number of transspinal and transcortical paired associative stimulation and assisted step training sessions is indicated for each participant. Motor evoked potential (MEP), transspinal evoked potential, and Medication was taken at similar times per day. xD, Times daily; M, Male; F, Female; C, Cervical; T, Thoracic; MVA, Motor Vehicle Accident; GSW, Gun Shot Wound.

a Indicates LR01 and LR11 are the same participant.

b Indicates LR04 and LR15 are the same participant.

c Indicates LR06 and LR12 are the same participant.

### Paired transspinal and TMS during locomotor training

The training protocol has been described in detail (27), and a summary is reported here. Transspinal stimulation was delivered before or after TMS during the mid-stance phase of robotic assisted step training. Transspinal stimulation and TMS were triggered based on threshold signals from a foot switch placed on the leg targeted by TMS. During assisted stepping, transspinal stimulation paired with TMS was delivered every 2–3 steps in four blocks of 10-min, with 2-min of no stimulation between each block. The total number of paired stimuli equalled 240 during each training session, resulting in 40-min of stepping with and 20-min of stepping without paired stimulation. Transspinal and TMS during assisted stepping were delivered at standing soleus TEP and MEP threshold, respectively. In the transspinal-TMS protocol, TMS was delivered at 58 ± 9.73 maximum stimulator output (MSO) and transspinal stimulation was delivered at 29.46 ± 15.15 mA across subjects. In the TMS-transspinal protocol, TMS was delivered at 58.83 ± 6.52 MSO and transspinal stimulation was delivered at 20.2 ± 11.41 mA across subjects.

Individualized interstimulus intervals (ISIs) for paired transspinal stimulation and TMS were estimated by adding 1.5 ms to the soleus (SOL) TEP latency and then subtracting the result from the SOL MEP latency (Equation 1). The 1.5 ms was added to the SOL TEP latency as the time required for synaptic transmission and conduction to the nerve root at the vertebral foramina (12, 29). In the participants that MEPs were absent (LR05 and LR14), TMS was set at the maximum tolerable intensity by the subject, while a 15.5 ms ISI was chosen based on data from a previous study (30).


(1) $$\begin{array}{l} \text{ISI }=\text{ SOLMEPlatency}-\left(\text{SOLTEPlatency}+1.5\text{ms}\right) \end{array}$$

One ISI was used for the same subject while reversing the order of triggering pulses to deliver TMS-transspinal or transspinal-TMS. In the TMS-transspinal PAS protocol, the ISI was designed so that corticospinal volleys arrived at corticospinal-motoneuronal synapses before motoneurons were depolarized by transspinal stimulation. The TMS-induced motor volleys are recorded from the upper thoracic level within 4 ms at a conduction velocity of ~62 m/s (31), while an average central motor conduction time to ankle muscles of ~15–19 ms in individuals with SCI has been reported (32). Moreover, when TMS precedes transspinal stimulation at ISIs ranging between 8 and 25 ms, TEPs and MEPs summate at the surface EMG suggesting for neuronal interaction at the spinal cord (21). The ISI in the transspinal-TMS protocol was designed so that excitation of posterior-root afferents and dorsal columns by transspinal stimulation could affect descending motor volleys at their site of origin. Given that stimulation of the spinal roots at the thoraco-lumbar spinal level elicits somatosensory evoked cortical potentials with latencies of roughly 12 ms in healthy individuals and 13–20 ms in individuals with spinal disorders (33–35), this evidence strongly supports transspinal stimulation-induced afferent regulation of corticospinal volleys at their site of origin at times as early as 12 ms.

Transspinal stimulation methods for both PAS protocols were similar to our previously published procedures (26, 27, 36). Using a constant current stimulator (DS7A, Digitimer Ltd., Hertfordshire, UK) cathodal transspinal stimulation was delivered *via* a self-adhesive electrode (Uni-Patch^TM^, 10.2 × 5.1 cm^2^, Wabasha, MA, USA) placed longitudinally at the T10 spinous process covering T10 to L1-2 vertebrae. Consistent stimulating electrode positioning between sessions were ensured by marking the electrode position with Tegaderm transparent film (3M Healthcare, St. Paul, MN, USA). The anode electrodes were placed either on the abdominal muscles or iliac crests based on comfort levels reported by each subject. Likewise, delivery of TMS was similar to our previously published methods (37–40). TMS was administered *via* a Magstim 200 stimulator (Magstim, Whitland, UK) with a double-cone coil (110 mm diameter) over the primary motor cortex. The coil was orientated to induce a posterior to anterior current. With subjects seated, we probed for the optimal stimulation position based on the minimum intensity needed to evoke soleus MEPs of at least 100 μV (41). The point was then marked on an EEG cap and the coil was held in place with a chin strap. The coil position was checked regularly during training sessions and the optimal stimulation position was re-confirmed, or adjusted, at the start of every new training week along with the MEP threshold.

### Locomotor training

Locomotor training was completed by all participants on the Lokomat 6 Pro^®^ for 5 days/week, 1 h/day for ~5 weeks. Treadmill speed, body-weight support (BWS), and leg-guidance force (LGF) of the robotic gait orthosis were adjusted over the course of the interventions to minimize knee buckling and ankle rolling during stepping (Table 2) similar to our previously published clinical trials on locomotor training for individuals with SCI (27, 42, 43).

**Table 2:** Training intervention parameters.

**Subject code**	**Speed (km/h)**		**BWS (kg)**		**LGF (%)**	
	**Before**	**After**	**Before**	**After**	**Before**	**After**
**Transspinal-TMS PAS and locomotor training**						
LR01^a^	2.2	2.6	21	15	70	50
LR04^b^	1.9	2.6	50	61	100	90
LR05	1.9	2.4	53	52	100	90
LR06^c^	2.1	2.2	53	26	90	64
LR07	2.2	2.4	43	43	80	70
LR09	1.5	1.6	68	59	100	95
**TMS-transspinal PAS and locomotor training**						
LR11^a^	2.3	2.4	17	6	65	38
LR12^c^	2.1	2.1	7	15	70	30
LR14	1.7	1.9	45	40	100	100
LR15^b^	1.7	1.7	61	43	80	96
LR20	1.9	1.6	16	23	70	46
LR21	1.6	1.9	36	33	100	100

The treadmill speed, body weight support (BWS), and leg guidance force (LGF) is indicated for each participant from the first training session (before) to the last training session (after).

#Indicates participants who completed both training groups following a 6-month washout between protocols.

a Indicates LR01 and LR11 are the same participant.

b Indicates LR04 and LR15 are the same participant.

c Indicates LR06 and LR12 are the same participant.

### Neurophysiological tests before and after stimulation and locomotor training

Following standard skin preparation, single bipolar differential electrodes (Motion Lab Systems Inc., Baton Rouge, LA) were used to record TEPs at rest and during assisted stepping from SOL, tibialis anterior (TA), peroneus longus (PL), medial gastrocnemius (MG), vastus lateralis (VL), rectus femoris (RF), medial hamstrings (MH), and gracilis (GRC) muscles from right and left legs. The electrodes were secured by Tegaderm transparent film. EMG signals were amplified and filtered at frequencies between 10 and 1,000 Hz, sampled at 20,00 Hz by a 1401 plus running Spike 2 (Cambridge Electronics Design Ltd., England, UK) during resting measurements or a National Instruments data acquisition board (National Instruments, Austin, Texas, USA) during stepping, and saved on a personal computer for offline analysis.

During standing with BWS on the Lokomat 6 Pro^®^, the right SOL TEP threshold was established, and corresponded to the lowest stimulation intensity that evoked a TEP response. The phase-dependent modulation of TEPs before and after each intervention were established during BWS assisted stepping across the step cycle that was divided into 16 equal time windows or bins with transspinal stimulation delivered at 1.3 multiples of the right SOL TEP threshold established during standing. Stimulation was triggered based on the threshold level of the right or left foot switch signals detecting heel contact and toe off (Motion Lab Systems Inc., Baton Rouge, LA, USA). Bin 1 corresponds to heel contact. Bins 9, 10, and 16 correspond approximately to stance-to-swing transition, swing phase initiation, and swing-to-stance transition, respectively. For each subject, at least five transspinal stimuli were delivered at each bin of the step cycle.

### Data analysis

The TEPs recorded from left and right ankle and knee muscles were measured as peak-to-peak amplitude during assisted stepping, averaged for each bin of the step cycle, and normalized to the homonymous maximal TEP observed across all phases of the step cycle. The mean amplitude of TEPs recorded at each bin of the step cycle was grouped based on time of testing (before and after training). Statistically significant differences before and after training were established with 2-way repeated measures analysis of variance (ANOVA) at 2 (time: before and after) times 16 (bins) levels followed by Holm-Sidak pairwise multiple comparisons to identify the bin number at which the TEP amplitude was significantly different before and after the intervention training. This analysis was performed for each TEP separately.

For each muscle, the background EMG activity for each bin was estimated from the mean value of the rectified and filtered EMG for a duration of 50 ms (high-pass filtered at 20 Hz, rectified, and low-pass filtered at 400 Hz), beginning 100 ms before transspinal stimulation. The mean amplitude of each TEP was plotted on the *y*-axis vs. the homonymous background activity normalized to the maximal control EMG on the *x*-axis, and a linear least-square regression was fitted to the data. The slope and intercept from the linear regression was grouped based on time of testing and statistically significant difference was established with a Student's *t*-test. In all statistical tests, significant differences were established at 95% of confidence level. Results are presented as mean values along with the standard error of mean (SEM).

## Results

### Motor neuron output before and after transspinal-TMS and locomotor training (Aim 1)

**Figure 1:**
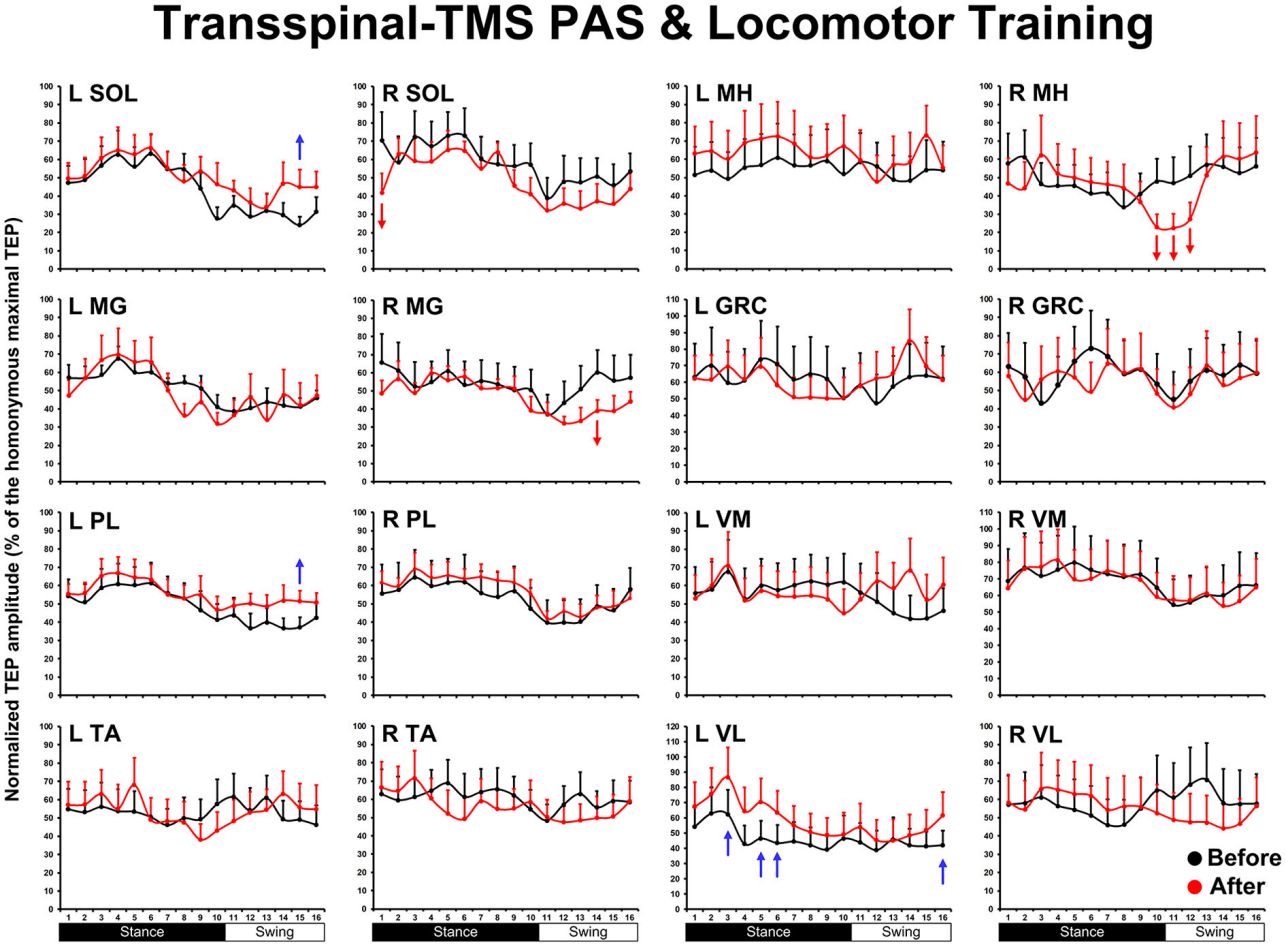
The mean amplitude of TEPs, from all subjects, recorded during assisted stepping from left and right muscles before (black) and after (red) transspinal-TMS PAS and locomotor training are normalized to the maximal homonymous TEP and plotted against the step cycle divided into equal 16 bins. Red arrows indicate depression and blue arrows indicate facilitation. Error bars indicate the SEM. SOL, soleus; MG, medial gastrocnemius; PL, peroneus longus; TA, tibialis anterior; MH, medial hamstring; GRC, gracilis; RF, rectus femoris; VL, vastus lateralis; TMS, transcranial magnetic stimulation; PAS, paired associative stimulation; TEPs, transspinal evoked potentials.

**Figure 2:**
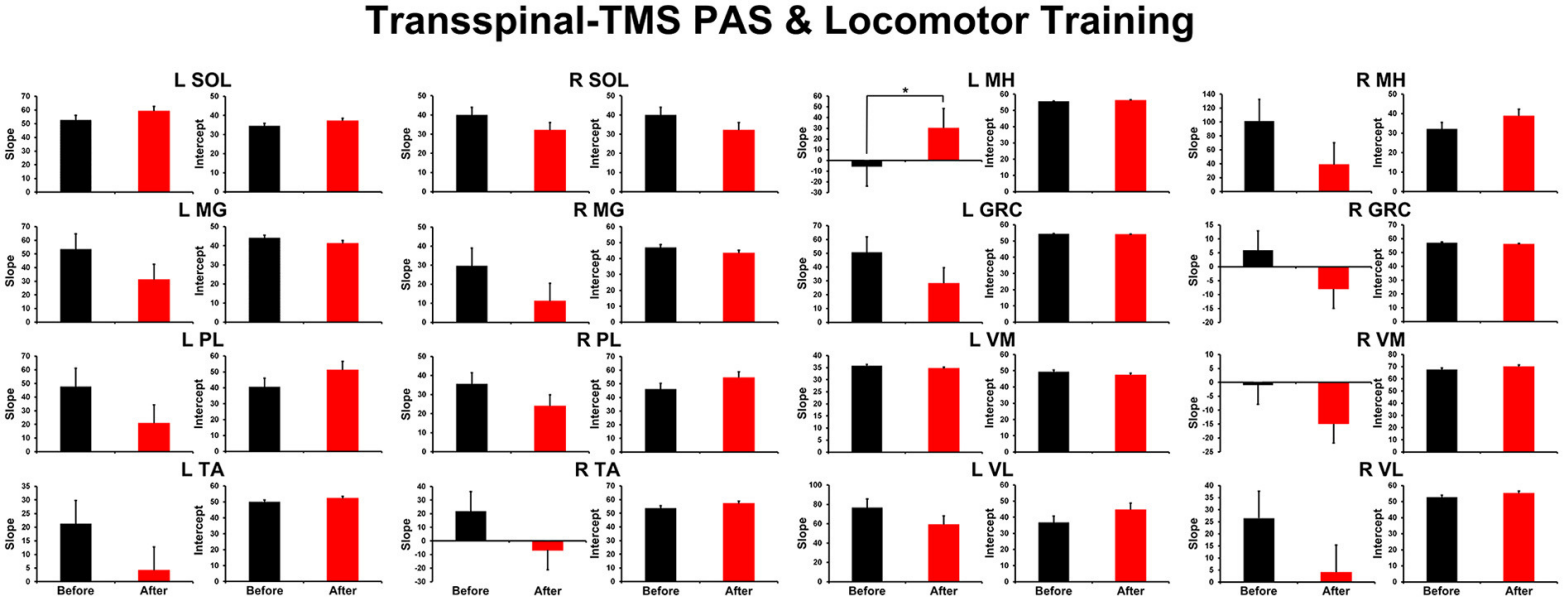
Overall amplitude of slope and intercept for TEPs recorded from left and right leg muscles. For each subject and muscle, a linear regression was applied to the normalized TEP amplitude plotted against the homonymous background EMG activity before and after transspinal-TMS PAS and locomotor training. An asterisk indicates a significant difference before and after intervention. Error bars indicate the SEM. PAS, paired associative stimulation; TEPs, transspinal evoked potentials.

The overall average TEP amplitudes during BWS assisted stepping recorded before and after transspinal-TMS and locomotor training from all muscles are presented in Figure 1. TEPs are grouped based on bin number of the step cycle and expressed as a percentage of the homonymous maximal TEP observed during assisted stepping across all step cycle phases. Significant differences of normalized TEP amplitudes as a function of time of testing were found in left SOL (F_1,189_ = 4.59, *P* = 0.033), left PL (F_1,188_ = 4.71, *P* = 0.031), right SOL (F_1,170_ = 8.34, *P* = 0.004), right MG (F_1,171_ = 6.2, *P* = 0.014), left VL (F_1,138_ = 12.28, *P* < 0.001), and right MH (F_1,138_ = 6.8, *P* = 0.013) muscles. The bin number that the TEP amplitude was significantly altered based on Holm-Sidak pairwise multiple comparisons is indicated in Figure 1 with a blue or a red arrow. These results support the increase of knee extensor motor output (left VL) during the stance phase and swing-to-stance transition and decrease of knee flexor motor output (right MH) during the mid-swing phase. The increased slope (computed from the linear regression between the normalized TEP amplitude and homonymous background EMG activity) of the left MH TEP (Figure 2) suggest an increased motoneuronal gain after training.

### Motor neuron output before and after TMS-transspinal and locomotor training (Aim 2)

**Figure 3:**
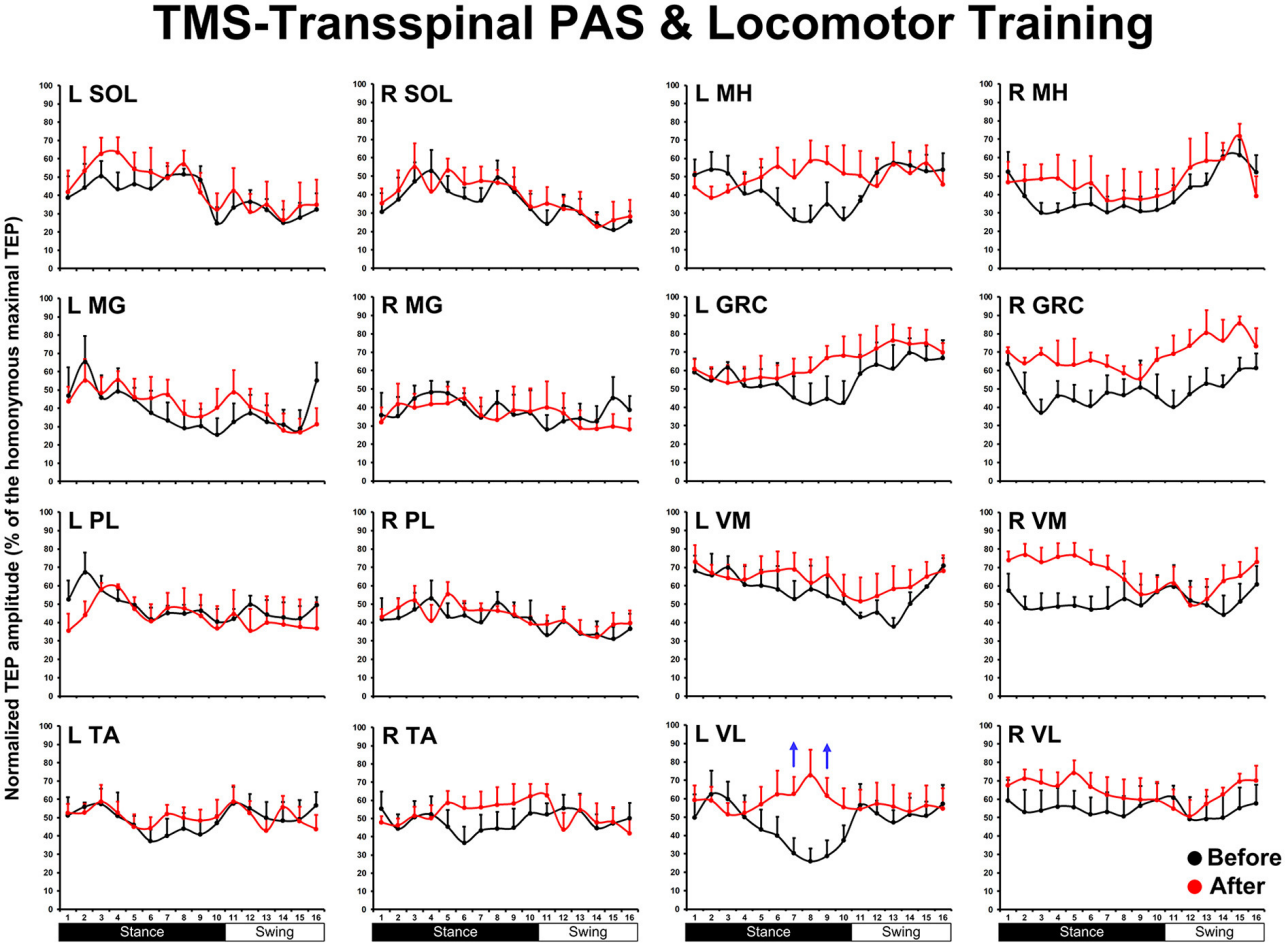
The mean amplitude of TEPs, from all subjects, recorded during assisted stepping from left and right muscles before (black) and after (red) TMS-transspinal PAS and locomotor training are normalized to the maximal homonymous TEP and plotted against the step cycle divided into 16 equal bins. Red arrows indicate depression and blue arrows indicate facilitation. Error bars indicate the SEM. SOL, soleus; MG, medial gastrocnemius; PL, peroneus longus; TA, tibialis anterior; MH, medial hamstring; GRC, gracilis; RF, rectus femoris; VL, vastus lateralis; TMS, transcranial magnetic stimulation; PAS, paired associative stimulation; TEPs, transspinal evoked potentials.

**Figure 4:**
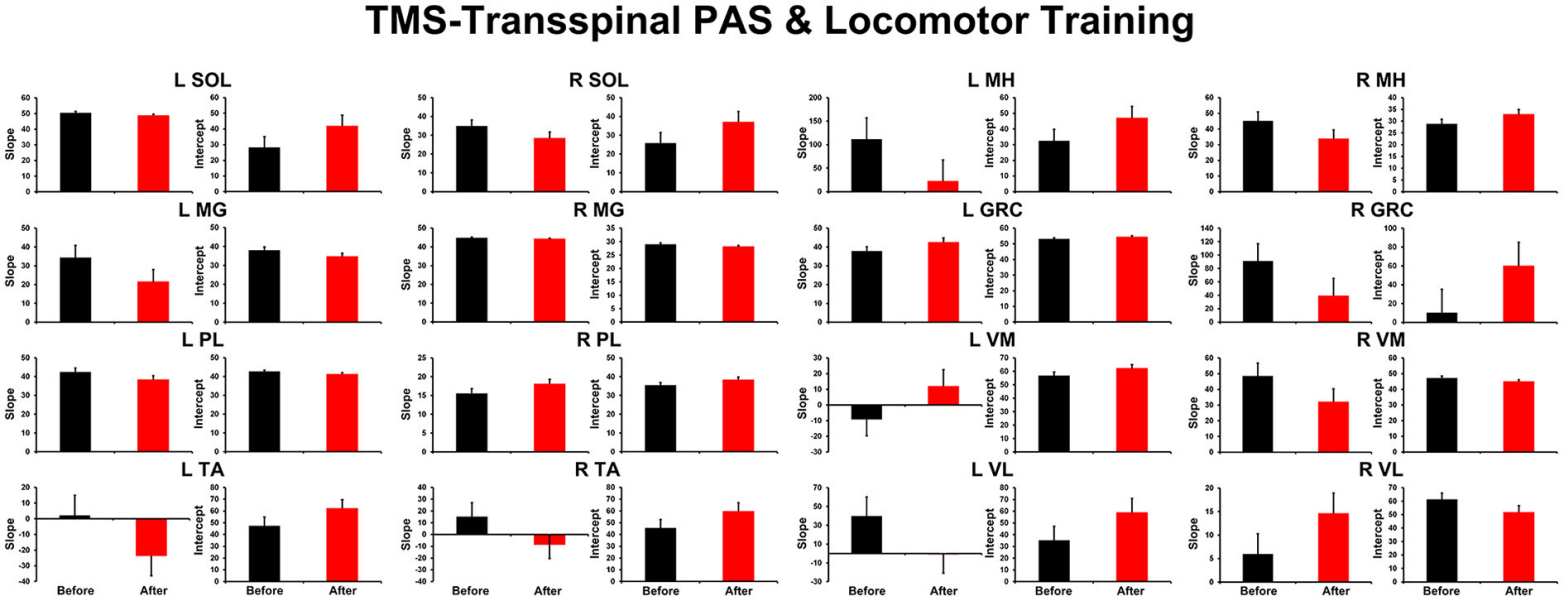
Overall amplitude of slope and intercept for TEPs recorded from left and right leg muscles. For each subject and muscle, a linear regression was applied to the normalized TEP amplitude plotted against the homonymous background EMG activity before and after TMS-transspinal PAS and locomotor training. An asterisk indicates a significant difference before and after intervention. Error bars indicate the SEM. TMS, transcranial magnetic stimulation; PAS, paired associative stimulation; TEPs, transspinal evoked potentials.

The TEP amplitudes during BWS assisted stepping recorded before and after TMS-transspinal and locomotor training from all muscles are presented in Figure 3. The average amplitude of TEPs from all subjects is indicated for each bin of the step cycle. TEPs are presented as a percentage of the homonymous maximal TEP observed during assisted stepping across all step cycle phases. Significant differences of normalized TEP amplitudes as a function of time of testing were found in left VL (F_1,127_ = 10.48, *P* = 0.002), left VM (F_1,112_ = 4.07, *P* = 0.046), right VL (F_1,160_ = 8.46, *P* = 0.004), and right GRC (F_1,160_ = 8.46, *P* = 0.004). For the remaining TEPs no significant effects as a function of time of testing was found (*P* > 0.05). These results suggest increase of motor neuron output in most proximal hip/knee muscles compared to ankle muscles. The slope (computed from the linear regression between the normalized TEP amplitude and homonymous background EMG activity) was altered after training for the left MH but not for the remaining muscles (Figure 4).

## Discussion

**Figure 5:**
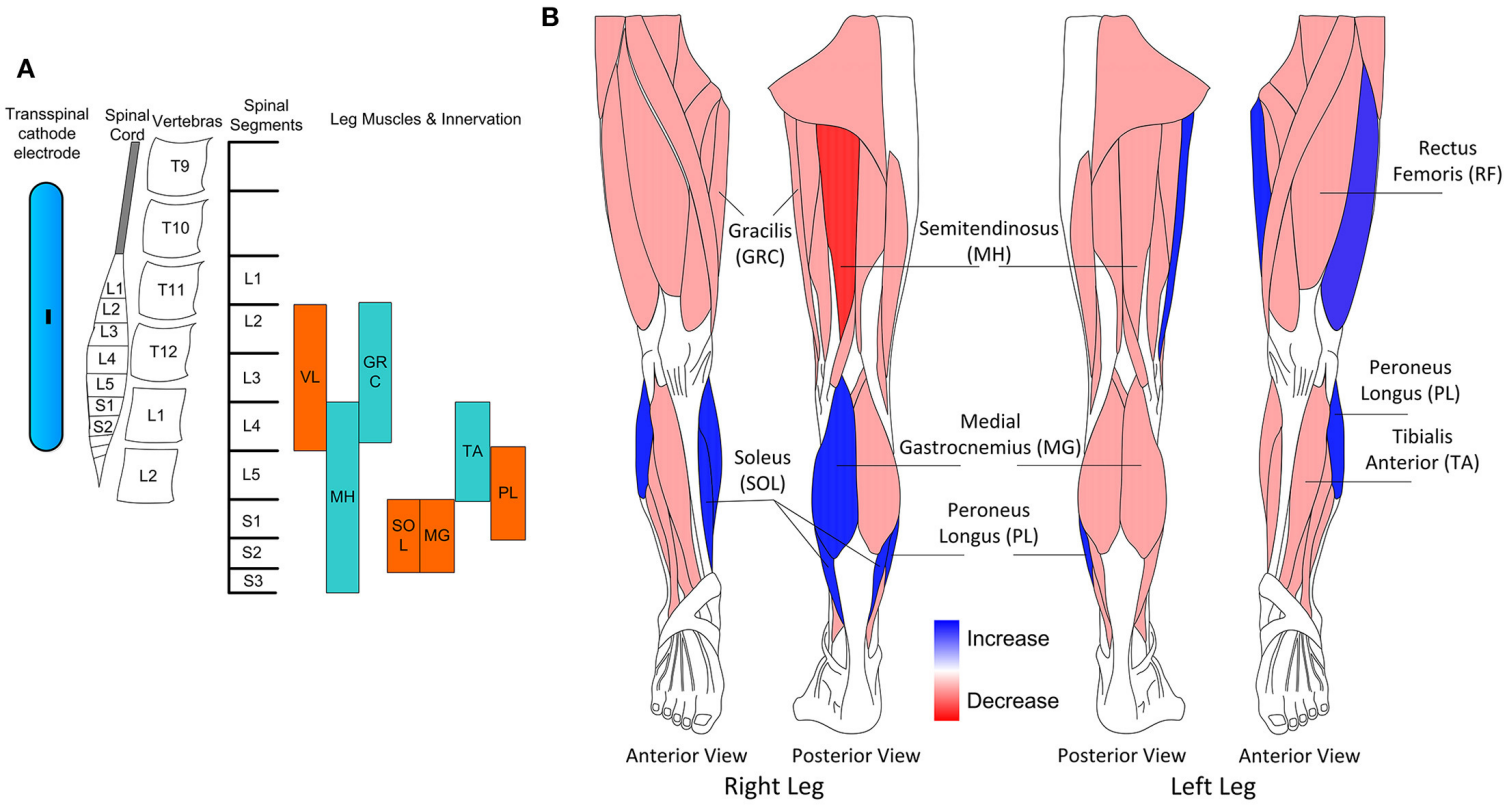
**(A)** Schematic presentation of the transspinal cathodal electrode relative to the spinal cord, vertebras, spinal segments, and innervation of muscles from which transspinal evoked potentials (TEPs) were recorded in this study. **(B)** Schematic presentation of the muscles in which increase or decrease of motor output was observed for both interventions.

In this study, we demonstrate changes in the motor neuron output after TMS and transspinal paired stimulation delivered during assisted step training in people with chronic SCI. After transspinal-TMS and locomotor training motor neuron output of knee and ankle muscles was modulated in a phase-dependent pattern, while after TMS-transspinal and locomotor training motor neuron output increased for knee muscles. These are novel findings because strengthening of motor neurons responsiveness occurred across multiple spinal segments after activity-based brain and spinal cord paired stimulation in people with chronic SCI. The location of the transspinal cathodal electrode along with the spinal levels and the innervation of muscles from which TEPs during assisted stepping were recorded in this study are shown in Figure 5A. The neuromodulation was centered from L1 to S2, but the effects were mostly pronounced in the extensor muscles (Figure 5B), linked possibly to sensory feedback (load and stretch) driven spinal locomotor networks (44), but consistent with our previous results when transspinal stimulation without locomotor training was delivered as an intervention to people with SCI (26). However, it is possible that this was the result of basing the ISI on the latency of the SOL MEP and TEP, supporting further for targeted neuromodulation of spinal motor neurons.

Both intervention protocols increased motor neuron output in individuals with chronic motor incomplete and complete SCI. It is possible that in both intervention protocols repetitive brain and/or spinal cord stimulation reorganized the receptive field of cortical and spinal motor neurons (45, 46). This may have involved expansion of the discharge zone of spinal motor neurons residing in the subliminal fringe and thus increasing the number of motor neurons being depolarized. Tonic excitation as a result of phasic motor activity increases activity of Renshaw cells found at subliminal fringe (47, 48), a condition that was apparent in our training interventions. Further, changes in intracortical circuits, depression of descending indirect waves, and activation of propriospinal neuronal networks after repetitive or conditioning transspinal stimulation in humans (39, 49), supporting reorganization of receptive fields and discharge zones of distributed neuronal networks.

Repetitive electromagnetic stimulation of the central nervous system during volitional motor activity potentiates corticospinal transmission in people with SCI (50) probably though long-term potentiation mechanisms occurring at spinal synapses of lower limb motoneurons (51). In both of our protocols, paired stimulation was delivered in presence of volitional motor activity during BWS assisted stepping. It is therefore possible that activity-dependent mechanisms of plasticity were utilized to alter the net motor neuron output, as expressed by the TEPs phase-dependent modulation of multiple muscles. For example, locomotor training alone is known to utilize reorganizational mechanisms such as axonal sprouting, unmask previously inactive synapses, and form new synapses (9, 52), while at the circuitry level pre- and post- motoneuronal spinal inhibition return (43, 53–55). All these changes may be due to return of dormant physiological pathways that increase the number of motoneurons being activated (8–10).

Regarding the specific sites of action, in the transspinal-TMS and locomotor training protocol transspinal stimulation was delivered at times sufficient to enable descending motor volleys to be affected at the site of their generation by dorsal spinocerebellar and dorsal column fibers (56). This possibility is supported by the effects of paired peripheral nerve and TMS stimulation on intracortical inhibition (57), and that somatosensory evoked cortical potentials with latencies of roughly 12 ms in healthy individuals and 13–20 ms in individuals with spinal disorders are reported following stimulation of spinal roots at the thoraco-lumbar spinal level (33–35). In the transspinal-TMS and locomotor training protocol, extensor VL, SOL, and PL motor neuron output increased and decreased for flexor MH, suggesting a more physiological regulation of motoneuron recruitment. This may be linked to altered cortical control of spinal inhibitory interneurons like the reciprocal Ia interneurons, but more research is needed to delineate potential neuronal mechanisms.

In the TMS-transspinal protocol the site of neuroplasticity is largely at the spinal cord neuronal networks because corticospinal descending motor volleys reached the spinal cord before or simultaneously with the transspinal stimulation. In this protocol, the amplitude of motor neuron output increased for left and right knee flexor or extensor muscles (Figure 3) suggesting altered responsiveness of multiple motoneuron pools, like what we have previously reported following repetitive transspinal stimulation alone in SCI (26).

Changes in the net motor output occurred with unaltered slope or intercept after training compared to before training (Figures 2, 4) suggesting that changes in the gain of the system was not due to changes in threshold excitability (58), but rather to the number of motoneurons being recruited enabling a more synchronized depolarization of motor neurons probably *via* LTP-like mechanisms. LTP can be produced following low frequency (0.2 Hz) stimulation (59), similar to what we used in this study.

### Limitations of the study

In this study, we did not include a control group receiving transspinal stimulation or locomotor training only. However, our previous studies using each intervention separately have clearly shown that our paradigm affected neuronal circuits in a similar way with concomitant depression of extensor and flexor reflex excitability (27, 28, 42). Further, due to notable differences between PAS protocols that utilize peripheral nerve and not transspinal stimulation, we cannot readily compare such studies. Last, we acknowledge that brain and transspinal stimulation during assisted stepping utilized as treatment in a rehabilitation setting would be challenging given the complexity of hardware—software arrangements and needs. However, our current protocol can be utilized at rest, and evaluate its priming effects on activity-based neurorecovery.

## Conclusion

In this randomized clinical trial, we delivered activity-based brain and spinal cord stimulation concomitantly with locomotor training. TEPs in people with SCI displayed step-related EMG amplitude modulation that was altered within the expected directions of the non-injured spinal state of locomotor EMG phase-dependent activity. Paired stimulation of the brain and spinal cord during locomotor training increased motor neuron output of the extensor muscles reflecting a repetitive finding that when neuromodulation is combined with locomotor training, sensory-input driven networks recover the most. Functionally, such changes can adjust locomotor EMG activity and thus improve quality of walking. The increased motor neuron output coincides with concomitant depression of spinal reflex excitability (27, 28), a phenomenon we observed also following transspinal stimulation alone (26, 36). In conclusion, activity-based brain and spinal cord stimulation can increase the net motor output improving motor ability in people with chronic SCI.

## Data availability statement

The original contributions presented in the study are included in the article/supplementary material, further inquiries can be directed to the corresponding author.

## Ethics statement

The studies involving human participants were reviewed and approved by Board of the City University of New York. The patients/participants provided their written informed consent to participate in this study.

## Author contributions

MK did conception and design of research, interpreted results of experiments, and wrote first draft of manuscript. TP, MZ, MG, EG, and MK performed experiments. MZ, MG, EG, and MK analyzed data. MZ and MK prepared figures. TP and MK edited and revised manuscript. All authors approved final version of manuscript.

## Funding

This study was supported by the New York State Department of Health (NYSDOH), Spinal Cord Injury Research Board (SCIRB), Wadsworth Center under Contracts C32248GG and C32095GG, and, in part, by the Eunice Kennedy Shriver National Institute of Child Health and Human Development (NICHD), National Institutes of Health (NIH) under award number R01HD100544 awarded to MK. Funding sources were not involved in study design, data collection, data analysis, data interpretation, or decision to publish.

## Conflict of interest

The authors declare that the research was conducted in the absence of any commercial or financial relationships that could be construed as a potential conflict of interest.

## Publisher's note

All claims expressed in this article are solely those of the authors and do not necessarily represent those of their affiliated organizations, or those of the publisher, the editors and the reviewers. Any product that may be evaluated in this article, or claim that may be made by its manufacturer, is not guaranteed or endorsed by the publisher.
